# Prebiotics and Probiotics: Healthy Biotools for Molecular Integrative and Modulation Approaches 2.0

**DOI:** 10.3390/ijms25094872

**Published:** 2024-04-30

**Authors:** Margarita Aguilera, Abdelali Daddaoua

**Affiliations:** 1 Department of Microbiology, Pharmacy School, University of Granada, 18071 Granada, Spain; maguiler@ugr.es; 2 Biosanitary Research Institute of Granada (IBS), 18014 Granada, Spain; 3 Institute of Nutrition and Food Technology “José Mataix”, Center of Biomedical Research, University of Granada, Avda. del Conocimiento s/n. Armilla, 18016 Granada, Spain; 4 Department of Biochemistry and Molecular Biology II, Pharmacy School, University of Granada, 18071 Granada, Spain

The current version (2.0) of this Special Issue corresponds to the continuity of the previous edition due to the significance and relevance of the topic. The purpose of this Special Issue is to highlight and further delve into the molecular mechanisms of prebiotics and probiotics, while providing a broad overview of their future prospects that will enrich this field. Therefore, the biotools introduced in the new edition now serve to continue the primary objectives of this research area, to intervene and enhance key physiological functions of the microbiota. Consequently, these biotools, incorporated in the updated version, have emerged as new resources for intervening in and enhancing a variety of host functions of the microbiota.

The significant role of beneficial gut bacteria in promoting overall human health is widely acknowledged. Specifically, gut bacteria in the colon perform numerous vital functions, including enhancing mineral balance, fortifying the intestinal barrier, regulating the immune response, and, consequently, contributing to human health and wellbeing. Moreover, probiotics and prebiotics are considered bio-products used as food or medicine. They play essential roles in maintaining a healthy balance of the gut microbial community, strengthening the immune system, and preventing severe intestinal and metabolic diseases. Significant progress has been made in the field of probiotics in recent decades. However, their mechanisms of action are still not fully described nor understood. Hence, the 2nd version of the present Special Issue presents a compelling array of reviews and original research articles centered on innovative biotools aimed at advancing the integrative management and enhancement of prebiotics and probiotics. In this regard, the studies underscore the broad range of results that are available to amplify the beneficial characteristics of these beneficial microorganisms and their bioactive products for future development of probiotics and prebiotics.

In this context, the work for Nogacka AM et al., 2024 contribution 1, which was performed in a fecal culture model and compared individuals with obesity to individuals with normal weight, demonstrated that various probiotics exhibit the capacity to influence the composition of the gut microbiota and its levels of antibiotic resistance genes (such as *tetM* and *tetO* genes). This correlation indicated that the incorporation of probiotics, mainly from the Bifidobacterium genus, was associated with a decrease in the levels of this taxon in the microbiota. Consequently, their interesting findings suggested the potential application of probiotics for reducing the levels of antibiotic resistance genes levels in the human gut microbiota, which constitutes an interesting target for the future development of new probiotics.

Similarly, Nelly Schropp et al., 2023 contribution 2, proposed that different bacterial strains exhibit preferences for specific prebiotics, such as inulin or xylooligosaccharides (XOSs), e.g., *Streptococcus salivarius* DSM 20067, which displays enhanced growth in the presence of prebiotic XOSs. In summary, while more strains demonstrated growth on both prebiotics tested, there was a preference for inulin, suggesting that XOSs were more selective compared to inulin. Moreover, Huixin Wu et al., 2023 contribution 3, investigated the effect of the impact of a diet supplemented with inulin on the prevention of estrogen receptor-negative mammary carcinoma using a transgenic mouse model. Their findings revealed that inulin supplementation effectively suppressed tumor growth and notably prolonged the onset of tumors in the transgenic mouse model. This effect was attributed to alterations in gut microbial composition and an elevation in plasma propionic acid levels, which were shown to modulate the expression and enzymatic activities of HDACs and DNMTs, thereby influencing cell proliferation and survival pathways. In summary, this study suggested that modulating the microbial composition of the gut through an insulin-supplemented dietary intervention may be a promising strategy for breast cancer prevention.

At the same time, the findings from Byung Chull An et al., 2023 contribution 4, clearly confirmed that a novel probiotics-derived protein, P8, effectively inhibits the progression of colorectal cancer (CRC). Hence, P8 was observed to directly interact with GSK3β, leading to the suppression of cell proliferation, and the P8-derived anti-proliferation signal pathway was also investigated, but the mechanism was still unclear. Similarly, a novel drug delivery system based on probiotics, *Pediococcus pentosaceus* SL4 (PP-P8), was developed through genetic engineering and demonstrated successful secretion of P8 into the human intestines. Further, orally administered PP-P8 was found to be safe in rodent and marmoset models, suggesting its possible use in the treatment of patients with CRC alongside other therapeutic modalities, such as chemotherapy, radiation therapy, and targeted therapy.

While Julio Plaza-Diaz et al., 2022 contribution 5 summarized that the role of consumption of new INN formula could improve the composition of the gut microbiota promoting healthier communities, more similar to infants exclusively receiving human milk before the weaning period, Mijangos-Trejo A et al., 2023 contribution 6 assumed that alterations in the gut–liver axis and changes in the gut microbiome are among the common risk factors for the pathogenesis of non-alcoholic fatty liver disease (NAFLD). Patients with this disease show increased bacterial overgrowth in the small intestine and impaired intestinal permeability. Hence, the benefits of probiotics and prebiotics as a therapeutic option for patients with NAFLD are clear. However, most of these studies were conducted in vivo. Therefore, more randomized clinical trials in humans are needed to recommend its use.

Ana Isabel Beltrán-Velasco et al., 2024 contribution 7, confirmed the beneficial use of *Lactiplantibacillus plantarum* in the symptomatological intervention of neurodegenerative diseases. The existence of gut microbiota dysbiosis was associated with systemic inflammatory processes present in neurodegenerative diseases, creating the opportunity for new treatment strategies. This work involved the modification of strains that constitute the gut microbiota to enhance synaptic function through the gut–brain axis. The results indicated that *Lactiplantibacillus plantarum*, either alone or in combination, improved the symptoms of neurodegenerative disease after the intervention.

Finally, it should be noted that Veronika S Mihailovskaya et al., 2023 contribution 8, have highlighted that commensal bacteriocin-producing *Escherichia coli* is of interest for possible use as a probiotic for selectively controlling the spread of pathogenic bacteria. This was highlighted through the assessments of biosafety and efficacy of two newly identified bacteriocin-producing *E. coli* strains, Q5 (VKM B-3706D) and C41 (VKM B-3707D), which were isolated from healthy farm animals. Likewise, the beneficial impacts of bacteriocin-producing *E. coli* are associated with the inhibition of enteropathogens through bacteriocins, competition for adhesion sites, and preservation of epithelial barrier integrity, which all effect improved the balance and natural functions of intestinal microbiota. This is achieved through the stimulation of mucin glycoprotein and antimicrobial protein secretion, as well as tight junction molecules, modulation of metabolic and immune processes, and other mechanisms. Thus, this work demonstrated that the short-term oral administration of *E. coli* Q5 and *E. coli* C41 to rats contributed to the preservation of intestinal homeostasis and provided protection from external influences, including infection with an enterotoxigenic beta-lactam-resistant *E. coli* strain. Given all the evidence, these two *E. coli* strains are promising candidates as probiotics for use in farm animals.

This Special Issue (2.0) focuses on significantly broadening our scientific understanding of the molecular mechanisms underlying novel and enhanced prebiotics and probiotics. Furthermore, it aims to offer a thorough and comprehensive overview of the latest advancements in this field alongside the evolving insights into global implications and potential transferability to various health- and food-related areas.

